# Heat-Induced Cross-Tolerance to Salinity Due to Thermopriming in Tomatoes

**DOI:** 10.3390/metabo14040213

**Published:** 2024-04-10

**Authors:** Tobias Körner, Ruven Gierholz, Jana Zinkernagel, Simone Röhlen-Schmittgen

**Affiliations:** Department of Vegetable Crops, Hochschule Geisenheim University, 65366 Geisenheim, Germanysimone.roehlenschmittgen@hs-gm.de (S.R.-S.)

**Keywords:** abiotic stress, thermomemory, *Solanum lycopersicum*, climate change, plant growth, plant development, secondary metabolites, fruit quality, fruit yield

## Abstract

Global plant production is challenged by unpredictable (a)biotic stresses that occur individually, simultaneously or staggered. Due to an increasing demand for environmentally friendly plant production, new sustainable, universal, and preventive measures in crop protection are needed. We postulate thermopriming as a suitable procedure that fulfills these requirements. Therefore, we performed thermopriming as a pre-conditioning on tomato transplants in combination with two subsequent salt stress treatments to evaluate their single and combined physiological effects on leaves and fruits with regard to plant performance, fruit yield and quality. We identified a cross-tolerance to salinity that was triggered by the preceding thermopriming treatment and resulted in an accumulation of phenols and flavonols in the leaves. Plant growth and fruit yield were initially delayed after the stress treatments but recovered later. In regard to fruit quality, we found an increase in carotenoid and starch contents in fruits due to thermopriming, while sugars and titratable acidity were not affected. Our results indicate that thermopriming can mitigate the impact of subsequent and recurrent stress events on plant performance and yield under production-like conditions.

## 1. Introduction

Plant protection is currently and will be confronted with various challenges associated with climate change, such as more frequent extreme weather phenomena [1]. Greenhouse production could be severely impaired, particularly by heat waves and other (a)biotic stress conditions. The tomato plant is one of the most relevant vegetable crops worldwide. Tomatoes are adapted to a wide range of environmental conditions, but current cultivars are moderately sensitive to salinity [2,3]. In greenhouse production, fertigated tomatoes are already cultivated under a relatively high EC of 2.5 dS m^−1^ that is close to the species-specific threshold for yield reduction [2,4]. Hence, additional (a)biotic stresses restrict and risk the production of tomatoes even under optimal growing conditions in greenhouses. Besides that, there are more global challenges in plant production, such as the scarcity of utilizable water for irrigation in various regions of the world [5,6]. By improving the overall plant tolerance to abiotic stress, saline water could be used in plant irrigation, and tomato plants could even be cultivated in moderately saline soils when there are no alternatives on-site, respectively.

The effect of single stress (e.g., heat or salinity) on plant physiology is well researched. However, studies on the combination of these stresses and their physiological effects on growth, fruit yield and quality of tomato plants are scarce, particularly when stresses are applied staggered and not simultaneously with recovery periods in between. Some studies indicate that tomato plants exhibit improved long-term performance when exposed to combined stresses as opposed to single stress factors [7,8,9,10]. In a previous study, we already determined the potential of thermopriming as a method for pre-conditioning tomato transplants to induce a (thermo-)tolerance which may help plants cope more effectively with future stress events [11]. This (thermo-)tolerance can be attributed to the memory that plants can acquire after experiencing abiotic or biotic stress during their life cycle [12]. In response to stress, plants synthesize and accumulate protective metabolites such as phenols and flavonoids in their leaves and fruits for protection against inevitable oxidative stress [8,13] as well as phenolic compounds under increasing salinity [14,15]. The exposure to high temperatures leads to the formation of reactive oxygen species in plant tissues, which impair the photosynthetic electron transport chain and consequently growth processes [16,17]. An abiotic pre-treatment of plants, such as priming, can therefore activate the plant’s defense and, thus, protect plants against other environmental stresses [18,19] such as salinity [9]. Various approaches can be used to evaluate the plant’s physiological reaction to thermopriming and recurrent stress conditions. For example, non-invasive sensors can detect changes in the content of leaf compounds (by the determination of vegetation indices) and the photosynthetic efficiency (e.g., electron transport rate and stomata conductance) [20,21]. Hence, priming of transplants can be used to intentionally trigger a (cross-)tolerance in plants [22]. In this study, we aim to confirm that a controlled, sub-lethal heat treatment (thermopriming) in early plant development can induce a cross-tolerance against salt stress and thereby prove the usefulness of thermopriming in tomato production. Therefore, we evaluate the effect of thermopriming with two subsequent salt stresses on i. plant growth and yield, ii. leaf compounds, and iii. fruit quality. This measure enables plant producers to grow hardened plants that are ‘naturally’ more tolerant to multiple stresses and thus are able to avoid fruit yield losses. On a global scale, (thermo-)priming may allow tomato production under extreme growth conditions (such as high salinity) by using water with moderate salinity for irrigation. This could increase the cultivation area and sustain high productivity levels to ensure and safeguard food production [2].

## 2. Materials and Methods

### 2.1. Experimental and Priming Conditions

In 2023, a 20-week lasting experiment was conducted at Geisenheim University (Geisenheim, Germany) from 12 February to 6 July to determine the growth and yield performance of truss tomato plants (*Solanum lycopersicum* L.) var. Adeleza (Enza Zaden Deutschland GmbH & Co. KG, Dannstadt-Schauernheim, Germany) that were thermoprimed beforehand and subsequently exposed to salt stress twice (Table 1). Priming was applied in form of a heat shock as ‘thermopriming’ in climate chambers (Fitotron^®^ HGC 0714, Weiss Technik GmbH, Reiskirchen, Germany) at 40 °C for 90 min according to Körner et al. [11] one week after sowing for seven consecutive days (Table 2). The plants were initially sown in multipot plates ‘HerkuPak D 77’ (Herkuplast Kubern GmbH, Ering/Inn, Germany) in the peat substrate ‘ORANGE Pikier’ (PATZER ERDEN GmbH, Sinntal-Altengronau, Germany). After thermopriming at BBCH 12 [23], they were potted in the peat substrate ‘ORANGE Topf’ (PATZER ERDEN GmbH, Sinntal-Altengronau, Germany) to be temporarily cultivated in 10 cm diameter pots. These pots were arranged in completely randomized blocks on tables in greenhouse chambers where they were kept for 22 days with a temperature of 22 °C during the day and 18 °C at night. During that period, transplants were treated once (7 March, 7 days after priming, DAP) or twice (21 March, 21 DAP) with salt stress in form of 100 mL 200 mM NaCl (EC: 20 dS m^−1^) or with 100 mL tab water as control. One day after the final salt treatment, 36 days after sowing (DAS), transplants were planted in six rows (two outer rows as borders without treatments) in substrate ridges (Einheitserde SP Topf grob, PATZER ERDEN GmbH, Sinntal-Altengronau, Germany) in a different greenhouse until 6 July. The experimental design comprised four completely randomized blocks with eight plants per parcel/treatment (n = 8) and one additional block on each side with border plants. One plant—at the end of each parcel—was excluded from measurements to avoid bias caused by the surrounding parcels treated differently. Hence, n = 6 experimental plants per parcel were used for measurements and sample taking.

In the climate chambers, the multipot plates were watered once daily after sowing without the addition of fertilizer. Following emergence, plants were fertigated once per day with 0.5% ‘Ferty 2 mega’ (Hauert HBG Dünger AG, Grossaffoltern, Switzerland) until the transplants were planted into soil. Afterwards, the plants were fertigated as follows:

- first two weeks: 0.122 g N m^−2^ d^−1^ (FertyBasis1/Ca(NO_3_)_2_/NH_4_NO_3_) and 0.2 g K_2_O m^−2^ d^−1^,
- next two weeks: 0.244 g N m^−2^ d^−1^ (FertyBasis1/Ca(NO_3_)_2_/NH_4_NO_3_) and 0.4 g K_2_O m^−2^ d^−1^,
- consecutive weeks: 0.366 g N m^−2^ d^−1^ (FertyBasis1/Ca(NO_3_)_2_/NH_4_NO_3_) and 0.6 g K_2_O m^−2^ d^−1^.

Irrigation was automatically regulated by tensiometers depending on the water demand of the control.

### 2.2. Growth and Yield Parameters

In this study, the following vegetative growth parameters were measured: plant height and number of leaves (principal growth stages defined by the BBCH-scale [23]), as well as generative parameters such as number of inflorescences, number of infructescences and yield. At the end of the experiment, the above-ground dry matter and accumulated fresh matter (under consideration of the defoliated senescent leaves) were determined. Plants were cut off on 1 June (16 weeks after sowing) due to space constraints (limited cultivation height) in the greenhouse.

Infructescences were reduced to six fruits per truss, as recommended by the cultivar’s breeder. Trusses were harvested twice per week—at the beginning and end of each week—starting at the end of May. After assessing fruit weight and yield per plant, fruits of early (third truss per plant), intermediate (fifth truss) and late (seventh truss) trusses were measured using the spectrophotometer CM-700d (Konica Minolta Business Solutions Europe GmbH, Langenhagen, Germany). Under consideration of gloss, three equatorial measurements were taken on each of the six fruits to determine their average coloration.

### 2.3. Leaf Compound Analysis

In young (freshly formed, already fully unfolded true leaves) and the oldest primary true (mature) leaves, the total chlorophyll content (TCC), total carotenoid content (TCarC), total anthocyanin content (TAC; expressed as cyanidin-3,5-O-diglucosid equivalents, CyEs), total phenolic content (TPC; expressed as gallic acid equivalents, GAEs), and the flavonoid content (FC) were colorimetrically measured 1, 3, 4, 5, 9 and 13 WAP (weeks after priming) by Infinite M200 microplate reader with Magellan 7.2 software (Tecan Group Ltd., Männedorf, Switzerland) according to Dörr et al. [24]. The FC was determined by two using two distinct procedures for i. flavanols and flavones luteolin (FC_Quercetin_; expressed as quercetin equivalents, QEs), and ii. rutin, luteolin, and catechin (FC_Catechin_; expressed as catechin equivalents, CEs) [25]. Three technical (undiluted) replicates were measured for each sample. Subsequently, the mean was calculated to minimize technical bias stemming from the microplate reader.

Stomatal conductance of water vapor (g_sw_) and electron transport rate (ETR) were assessed by LI-600 (LI-COR Environmental, Lincoln, NE, USA) to evaluate the plant stress in response to the experimental treatments. Therefore, one abaxial measurement on one young leaf per experimental plant was carried out in the morning, shortly after sunrise.

### 2.4. Fruit Compound Analysis

Fruit coloration (L*a*b* values) of early, intermediate, and late trusses was measured after harvest. Based on the L*, a* and b* values, color indices, such as hue angle (Hue), color index, color difference with true red and a*/b*, were calculated in accordance with López Camelo and Gómez [26].

Afterwards, all six fruits per truss were vertically cut into eight parts for three different types of fruit analysis;

1. For colorimetric analysis, mixed samples consisting of one-eighth of each fruit were immediately frozen in liquid nitrogen and stored at −80 °C;
2. For the analysis of ascorbic acid, approximately 100 g of one eighth of each fruit were mixed, weighed in a vessel, filled up with 200 g of 1% (*w*/*v*) aqueous oxalic acid dihydrate (≥99.5%; Carl Roth GmbH + Co. KG, Karlsruhe, Germany) and frozen at −20 °C;
3. For the determination of titratable acidity, the remaining fruit parts were frozen in liquid nitrogen and stored at −80 °C.

The analytical method for ascorbic acid (ASC) was adapted from Abe-Matsumoto et al. [27] and Tanner and Brunner [28]. Samples that were not yet fully thawed were mashed with a hand blender and centrifuged at 15 °C for 12 min. Subsequently, 25 mL of aliquots were weighed-in, and 15 mL of 10% sulphuric acid (Carl Roth GmbH & Co. KG, Karlsruhe, Germany) as well as a spatula tip of potassium iodide (≥99.5%; Carl Roth GmbH & Co. KG, Karlsruhe, Germany) were added. Samples were then analyzed as duplicates using an iodid-iodate (*v*/*v*) standard solution (1/128 mol I_2_ L^−1^—1/64 N; Carl Roth GmbH & Co. KG, Karlsruhe, Germany) with the Metrohm 702 SM Titrino titrator, Metrohm 730 Sample Changer and a double Pt sheet electrode (Metrohm 6.0309.100) controlled by the software Tiamo 2.5 (all obtained from Deutsche METROHM GmbH & Co. KG, Filderstadt, Germany). As standard solution was used 100 ± 0.1 mg L(+)-ascorbic acid (L-ASC) (≥99%; Carl Roth GmbH & Co. KG, Karlsruhe, Germany) was solved in 100 mL 1% (*w*/*v*) aqueous oxalic acid dihydrate to obtain a concentration of 1 mg mL^−1^. The concentrations of ASC were expressed in mg kg^−1^ by using the following equations:

ASC [mg L^−1^] = (c(I_2_) × M(L-ASC) × 1000 × V(I_2_))/V(aliquot)

mass Factor = (m(fruit) [g] + m(oxalic acid) [g])/m(fruit) [g]

ASC [mg kg^−1^] = ASC [mg L^−1^] × mass factor

The colorimetrically measured fruit compounds (TCarC, TAC, TPC and FCs) were determined according to Dörr et al. [24]. However, in contrast to leaf samples, only 5 mg of freeze-dried, grounded samples (one mixed sample per truss at each stage of harvest) were extracted with 1 mL of methanol (≥99%; Carl Roth GmbH + Co. KG, Karlsruhe, Germany) and then measured with three technical replicates.

For analysis of titratable acid adapted from DIN EN 12147 [29] and OECD guidelines [30], 150 mL of DI water was added to 50 g of thawed puree. After 60 min (stirred every 15 min), the solution was filtrated with type 520b technical filter paper (creped; Hahnemühle FineArt GmbH, Dassel, Germany). Then, 50 g of the aliquot was weighed-in, followed by the addition of 150 g of DI water. The mixture was eventually placed on a sample changer. The titration was conducted with the Metrohm electrode plus (Deutsche METROHM GmbH & Co. KG, Filderstadt, Germany) and the Metrohm 702 SM Titrino titrator. Sodium hydroxide (*v*/*v*) standard solution (0.33 mol NaOH L^−1^—0.33 N; Carl Roth GmbH & Co. KG, Karlsruhe, Germany) was used as reagent and 5% (*w*/*v*) aqueous citric acid (CA, ≥99.5%; Carl Roth GmbH & Co. KG, Karlsruhe, Germany) as standard. The samples were titrated until pH 8.1 was reached. The total content of titratable total acid (TA; expressed as CAEs, citric acid equivalents) was calculated as follows:

EP2 = volume (V) of 1/3 N NaOH until pH 8.1

Z = EP2 × 21.34 (specific factor for citric acid)

P = (W_sample_ × W_filtrated aliquot_) × W_sample_ + V_DI water_)

TA = Z/P

where W is the weight of the sample or filtrated aliquot.

Furthermore, sugars, such as glucose and fructose, and starch were determined in the following steps adapted from Gomez et al. [31] and Zhao et al. [32]:

4. Sugar Extraction:

For the extraction of sugars, 10 mg of freeze-dried and grounded fruit were weighed in 2 mL test tubes. A total of 1.5 mL of 80% ethanol were added (diluted from ≥99.5%; Carl Roth GmbH + Co. KG, Karlsruhe, Germany), mixed and incubated at 80 °C for 15 min in a dry bath. Samples were then centrifuged at 7000 rpm for 5 min. The supernatant was stored in 15-mL test tubes. These extraction steps were repeated twice to obtain an extract volume of 4.5 mL in total. Finally, the pellets were dried at 60 °C in a dry bath for starch analysis.

5. Preparation of Standard Solutions:

For the determination of the sugar content in fruits, standards with a known concentration of glucose were used. The standard stock solution was prepared for 1 mg glucose mL^−1^ by dissolving 50 mg of D(+)-glucose monohydrate (≥99.5%; Carl Roth GmbH + Co. KG, Karlsruhe, Germany) in 50 mL double deionized (DI) water. Thereof, dilutions were made with 0.025, 0.05, 0.25, 0.5 and 1 mg glucose mL^−1^. The absorbance of the glucose standards was measured at 340 nm with the Infinite M200 microplate reader to calculate the standard curve equation and thus the sugar content of fruit samples.

6. Preparation of Buffers and Enzymes:
  - Citrate buffer. For 250 mL buffer, 8.625 g of citric acid (≥99.5%; Carl Roth GmbH + Co. KG, Karlsruhe, Germany) and 11.375 g tri-sodium citrate dihydrate (≥99%; Carl Roth GmbH + Co. KG, Karlsruhe, Germany) were dissolved in a 250 mL volumetric flask with DI water. The pH was adjusted to 4.6 with 2 mol L^−1^ sodium hydroxide solution. The buffer was stored at 4 °C.
  - Tris buffer. For making 250 mL tris(2-hydroxyethyl)ammonium chloride (tris buffer), 43.75 g triethanolamine hydrochloride (≥99%; Carl Roth GmbH + Co. KG, Karlsruhe, Germany) and 0.78 g magnesium sulphate heptahydrate (100%; Carl Roth GmbH + Co. KG, Karlsruhe, Germany) were dissolved in a volume of 250 mL DI water. The pH was adjusted to 7.6 with 5 mol L^−1^ sodium hydroxide solution. This buffer solution was also stored at 4 °C.
  - Tris-combi buffer. In a 15 mL test tube with 10 mL tris buffer, 100 mg sodium hydrogen carbonate (≥99.5%), 100 mg adenosin-5′-triphosphate disodium salt (≥98%), and 20 mg NADP disodium salt (≥85%; all obtained from Carl Roth GmbH + Co. KG, Karlsruhe, Germany) were dissolved.
  - Hexokinase for glucose/glucose 6-phosphate dehydrogenase (HK/G6P-DH). For this buffer, 500 µL HK/G6P-DH (Roche Pharma AG, Basel, Suisse) was diluted with 500 µL DI water.
  - Phosphoglucose isomerase (PGI) fructose. For this buffer, 50 µL PGI (Roche Pharma AG, Basel, Suisse) was diluted with 950 µL DI water.
  - Amyloglucosidase (AMG). In 2 mL citrate buffer 5.6 mg AMG was dissolved.

The Tris-combi-buffer and all enzymes were prepared on the day of use.

7. Measurements of Glucose and Fructose:

Round-base 96-well microplates (Sarstedt, Nümbrecht, Germany) were used for the determination of sugar contents. For each sample, 20 µL of the extract was pipetted with three replicates. The microplates were heated to 60 °C for 10 min to evaporate the ethanol. After concentrating the extract, 20 µL of DI water was added to dissolve the sugars again. Following, standards and blanks were pipetted to their respective wells. In the next step, 100 µL of tris-combi buffer was added to each well before measurements were conducted at 340 nm with the Infinite M200 microplate reader. After the first measurement, 10 µL of HK/G6P-DH were added to each well. Then, the plate was placed on a shaker at 30 °C at 200 rpm for 30 min. A second measurement was conducted at 340 nm to analyze the glucose content. Afterward, 10 µL of PGI were added to the wells and incubated at 30 °C and 200 rpm on a shaker for 30 min. After incubation, the final measurement of fructose was performed at 340 nm. The glucose and fructose contents were calculated as follows:

Sugar content = [((A_2_ − A_1_) − A_blank_) × C × V/W] × 100

where A is the absorbance (A_1_: initial A; A_2_: final A; A_blank_: measurement of a blank solution with reagents that did not contain the sample), *C* the constant that was determined with a known standard concentration, V the final volume of extracted solution, and W the weight of the sample.

8. Starch Measurements:

The dried pellets (from the sugar extraction) were resuspended with 500 µL 0.5 mol L^−1^ sodium hydroxide solution, mixed, centrifuged at 7000 rpm for 30 s, and then incubated at 85 °C for 60 min in a dry bath. After cooling down to room temperature, the samples were neutralized with 15 µL of 100% acetic acid (Carl Roth GmbH + Co. KG, Karlsruhe, Germany) and filled up with 485 µL of DI water to a total volume of 1 mL before being centrifuged again at 5000 rpm for 5 min. The supernatant of samples, standards, and blanks was pipetted with 10 µL and three replicates into round-based 96-well microplates (Sarstedt, Nümbrecht, Germany). Then, 20 µL of AMG was added to each well, and the plate was incubated at 60 °C for 60 min (without mixing). After cooling down to room temperature, 100 µL of tris-combi buffer was added to each well. The samples were initially measured at 340 nm. For measuring the glucose content (after starch degradation), 10 µL of HK/G6P-DH was added and incubated at 30 °C and 200 rpm for 30 min before the final measurement at 340 nm was conducted. The starch content was determined indirectly, according to the previous formula, through the degradation of starch into glucose.

### 2.5. Multiple Factor Analysis

Multiple factor analysis (MFA) was separately computed for the leaf pigments and compounds as well as the fruit pigments and compounds, respectively, with ‘treatment’ as the active variable. For leaf indices and compounds, the supplementary variables ‘leaf age’ and ‘date of measurement’ were chosen. Regarding fruit compounds, MFA was applied to the colorimetrically measured compounds (TCarC, TAC, TPC, and FCs), the glucose, fructose, starch, and ASC content, as well as the titratable acidity as quantitative variables. Supplementary variables such as fruit coloration (Hue, color index, color difference with true red, and a*/b*) and stage of harvest were included in the analysis.

### 2.6. Data Analysis

The statistical analysis was conducted using R (version 4.2.2) with a linear mixed-effects model for ANOVA (α = 0.05; car–package, version 3.1.1). Post hoc analysis was carried out using estimated marginal means (EMMs, α = 0.05, Tukey-adjusted; emmeans–package, version 1.8.4.1) combined with the cld–function (multcomp–package, version 1.4.23) for the display of letters indicating significant differences in pairwise comparisons (α = 0.05). Therefore, the lmer–models (lmerTest–package, version 3.1.3) were specified depending on the measurement date, or in the case of the fruit parameters, the stage of harvest and the fruit coloration as covariates, and the random effects of the completely randomized block and repeated measurements. In the special case of fruit parameters that used a fruit color index in their linear model, a model comparison between models with different color indices was conducted using the performance-package (version 0.10.2) to identify the best model for each fruit parameter. In consequence, the color index was only used for TPC and ascorbic acid content, whereas the color difference of true red was chosen for all other fruit parameters. Additionally, MFA was computed with the factoextra–package (version 1.0.7). Therefore, the colorimetrically determined leaf and fruit compounds, as well as the vegetation indices, were previously cleared of outliers that were detected using the interquartile range criterion. The same procedure was applied to g_sw_ and ETR. Finally, plots were created by ggplot2–package (version 3.4.1).

## 3. Results

### 3.1. Thermopriming Did Not Impair Plant Growth and Yield Performance after Subsequent Stress

Plant growth and development were initially affected by thermopriming but mainly by the subsequent salt stress applications (Table A1). After priming, the plant growth (number of leaves) of thermoprimed plants was delayed. Nevertheless, plants reached the same growth stage as non-primed plants at the time of the first salt stress application. After the first stress application, plant growth remained similar between treatments. Although salt-stressed plants generally displayed delayed growth by a decreased number of leaves (−6%), which was even more pronounced after the second salt stress treatment (up to −10% in primed, twice salt-stressed plants). However, primed and non-primed plants coped similarly with salt stress. Therefore, thermopriming did not prevent a delay in plant growth after plants were exposed to subsequent stresses. Plants that were solely thermoprimed did not differ in growth from plants in the (non-primed) control for the whole experimental duration (after an initial delay). These results illustrate that plants recovered quickly from the heat stress caused by thermopriming. However, they did not cope well with the recurring salt stress, which caused severe delays still noticeable eight weeks after the application of the second salt stress. Furthermore, we did not find any differences between treatments in the final measurements of fresh and dry matter (Table A2).

After the first salt stress, the salt-stressed plants displayed a decreased g_sw_, proving the first stress was intense enough to trigger a stress response (Table A3). Primed and salt-stressed plants demonstrated the strongest decrease in g_sw_ (−17%) in comparison to the control. After the second salt stress application, the g_sw_ of primed, or non-primed and non-stressed plants was similar but increased compared to the plants that were exposed to salt stress twice for at least two weeks. Hence, thermopriming as a single treatment only had an initial effect on g_sw_ that was balanced out soon after the application, whereas salinity had a predominant effect on g_sw_. Starting one month after the second stress application, all treatments were similar in their g_sw_. For ETR, the treatments did not show consistent effects. After the second salt stress, however, control plants had a lower ETR compared to plants that were salt-stressed twice or thermoprimed.

**Figure A1:**
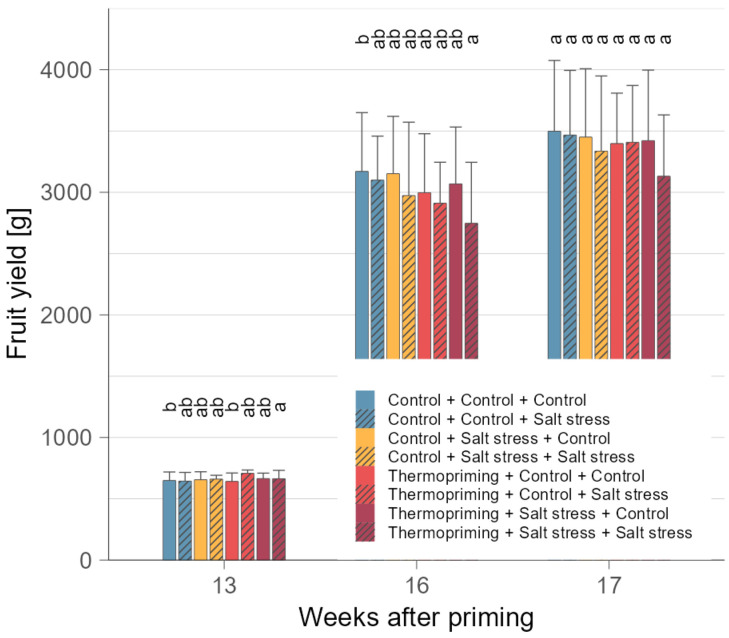
Accumulated fruit yield weeks after priming differentiated for the eight treatments. The different letters indicate significant differences (ANOVA and EMMs post hoc; α = 0.05) between groups at the same week after priming.

Thermopriming and subsequent stresses initially delayed the generative development one week after the second salt stress (4 WAP) in comparison to the control (Table A1). Then, at 7 WAP, thermoprimed but not salt-stressed plants did not have a decreased number of inflorescences anymore. At 10 WAP, the thermoprimed and twice-stressed plants caught up to the control and temporarily even displayed an increased number of inflorescences (+18%). In the following weeks, plants from all treatments had a similar number of inflorescences. Similarly, the number of infructescences and fruit yield initially decreased due to thermopriming and subsequent stresses (Table A1; Figure A1). Though, in the last weeks of the experiment at the end of June, the accumulated fruit yield of all treatments was aligned (Table A2).

Overall, growth and development of thermoprimed plants were initially delayed after priming, and this delay was further increased by the subsequent salt stresses. However, in consecutive weeks after priming, thermoprimed and twice-stressed plants developed similar to the other treatments and even exhibited a temporarily increased number of inflorescences (+18%). During the whole experimental period, salt-stressed plants displayed delayed development, while primed and non-primed plants coped similarly with salt stress. Thus, the preceding thermopriming did not prevent delayed plant development due to subsequent stress(es). Although fruit yield was initially reduced due to delays in the flower and fruit development stages caused by priming and subsequent stresses, it was eventually balanced out.

### 3.2. Thermopriming Decreased Acidity and Sugars, but Increased Starch and Carotenoids in Fruits

In terms of fruit quality, twice-stressed and previously thermoprimed plants displayed an accumulation of carotenoids in early inflorescences (+38%) compared to recurrently stressed non-primed plants, which had the lowest TCarC (Table A4). Besides that, no distinctive effects of thermopriming or subsequent salt stress treatments on the accumulation of pigments and secondary metabolites were found in fruits. The coloration of fruits at the time of harvest was recorded in order to consider the external state of ripeness as a decisive factor for quality differences. Moreover, potential interactions between thermopriming and the subsequent stress treatments were investigated but were not significant. In contrast to all treatments, only the primed and most stressed group was affected and displayed a decreased titratable acidity in late infructescences (−12%; Table A5). This observation was associated with a difference in the color of tomato fruits. There was not any effect on titratable acidity for early and intermediate infructescences or under consideration of all harvest stages. ASC was decreased in the early fruits of primed and subsequently stressed plants compared to primed plants that were not stressed. No effects were found on intermediate and late infructescences, respectively. Hence, increased stress levels did not lead to an accumulation of ASC in fruits. Moreover, glucose and fructose contents decreased in the early and intermediate infructescences of primed and subsequently stressed plants. The glucose and fructose contents of early infructescences were not affected by the ripeness (coloration) of fruits. Fruit ripeness only had an effect on intermediate fruits. Neither glucose nor fructose were affected by the treatments in late infructescences. Primed plants that were subsequently stressed had an increased starch content in fruits compared to non-primed plants. Thermopriming resulted in a higher accumulation of starch in late infructescences (+54%). Early and intermediate fruits did not differ in their starch content between all treatments.

Thermoprimed, twice-stressed plants displayed an accumulation of TCarC in early trusses (+38% compared to non-primed, twice-stressed plants with the lowest TCarC, or +15% to control) and a decreased titratable acidity in late infructescences (−12%). No distinctive effects on the accumulation of other secondary metabolites by thermopriming or subsequent salt stress(es) were found. Additionally, stress led neither to increased levels of ASC nor glucose and fructose but to an accumulation of starch in late infructescences.

**Figure 1:**
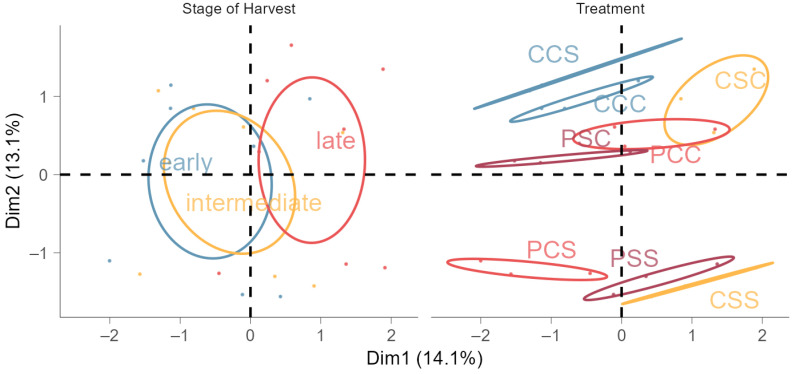
Multiple factor analysis of standardized primary and secondary fruit compounds for group means (specified by treatment and stage of harvest; displayed as points for three stages of harvest (early, intermediate, and late) and the eight treatments as confidence ellipses with β = 0.95). Treatments: P represents thermopriming, C control conditions (non-primed or non-stressed groups), and S salt stress (e.g., C–C–C: control conditions (non-primed) instead of priming conditions, non-stressed (C) at the time of the first salt stress, and non-stressed (C) at the time of the second salt stress). Multiple factor analysis was performed on the active variable treatment as well as the supplementary variable stage of harvest.

**Figure A2:**
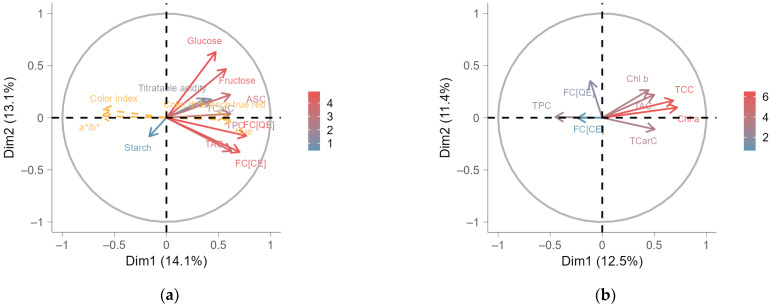
Multiple factor analysis of standardized primary and secondary (**a**) fruit and (**b**) leaf compounds (TCC: total chlorophyll content; Chl a: chlorophyll a; Chl b: chlorophyll b; TCarC: total carotenoid content; TAC: total anthocyanin content; TPC: total phenolic content; FC: flavonoid content (expressed as CEs, catechin equivalents, or QEs, quercetin equivalents); Dualex chlorophyll (Chl) index; Dualex flavonol (Flav) index; ASC: ascorbic acid) for group means (specified by (**a**) treatment and stage of harvest or (**b**) treatment, leaf age, and date). Multiple factor analysis was performed on the active variable treatment as well as the supplementary variables: (**a**) stage of harvest and color indices (yellow arrows) or (**b**) leaf age and date. The color gradient (from blue to red) indicates the contribution of the variables to the dimensions (Dim).

This negative correlation was also demonstrated by the MFA results (Figure A2a). In MFA, sugars (glucose and fructose) and FCs accounted for most of the variability among all the input variables. Glucose and fructose were correlated positively but displayed a negative correlation to the starch content in fruits. Accumulated TPC, FCs and TAC were positively correlated as well, but not with sugars, titratable acidity, or ASC. Regarding treatments (Figure 1), the control group and the non-primed, late-stressed group were clustered together. In the opposite quadrant, twice-stressed groups were clustered together—independently from the thermopriming pre-treatment. Thus, plants that were stressed twice were similar in their metabolites but showed a negative correlation to the control. Particularly thermoprimed groups were clustered separately in between. Consequently, groups that were previously thermoprimed responded differently to subsequent stress, although the predominant effects of the recurrent stress overlaid this effect. The harvest stage of fruits had an influence on the accumulation of metabolites in fruits. Moreover, the cluster of early and intermediate harvested trusses overlapped strongly, whereas late trusses only displayed a partially overlapping effect with the other stages of harvest.

### 3.3. Accumulated Phenols and Flavonoids in Leaves Indicate Cross-Tolerance to Salinity

In trend, lower TCC was found for mature leaves of thermoprimed plants in comparison to non-primed control plants for two weeks after the second salt stress (Table A7). This effect was significantly exhibited in thermoprimed and twice-stressed plants (−27%). After that, treatments did not differ in their TCC anymore. In accordance with mature leaves, young leaves also showed a decrease in TCC in response to thermopriming (Table A6). This was still apparent two months after the second salt stress treatment.

The TCarC in leaves was generally decreased in salt-stressed plants, whereas non-stressed groups that were either thermoprimed or non-primed did not differ. Young leaves of primed plants displayed a decreased TCarC after priming (−9%), but showed an increased TCarC one week after the first salt stress application in primed plants that were not salt-stressed in comparison to non-primed plants that were salt-stressed (Table A6). One week after the second stress, the control group had a higher TCarC compared to primed, once-stressed plants (up to −162%) and, most of all, primed, twice-stressed plants (−209%). After that, all treatments displayed a similar TCarC in young leaves.

A decreased TAC (−19%) was found in mature leaves of primed plants compared to the control group three weeks after thermopriming (Table A7). Simultaneously, the first salt stress resulted in a significant decrease of TAC in all treatments in comparison to the control (primed: −25%; non-primed: −36%). The second salt stress affected plants even more strongly, resulting in the lowest TAC in primed, twice-stressed plants (−19%) in comparison to the control and the highest TAC in mature leaves of non-primed, twice-stressed plants. One month after the second stress, TAC was similar between all treatments. At first, TAC in young leaves was neither affected by thermopriming nor the first salt stress (Table A6). After the second stress treatment, primed and twice-stressed plants had the lowest TAC in young leaves (−19%). Just one week later, however, they did not differ in TAC from the control and even showed an increased TAC compared to the primed group that was either non-stressed or stressed once.

**Figure 2:**
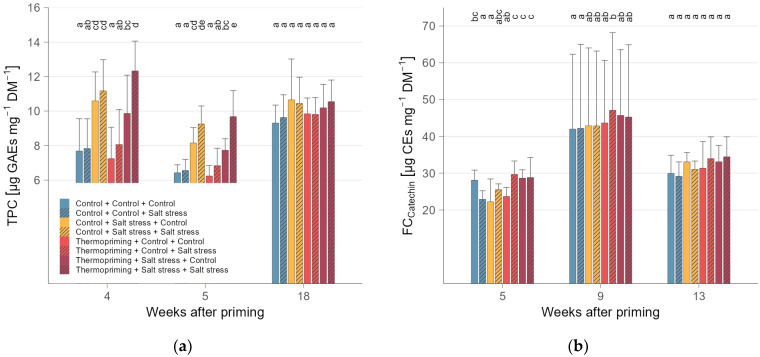
(**a**) Total phenolic content (TPC; expressed as GAEs, gallic acid equivalents) and (**b**) flavonoid content (FC; expressed as CEs, catechin equivalents), displayed by mean (columns) and standard deviation (whiskers), of tomato plants weeks after priming in (**a**) mature and (**b**) young leaves differentiated for the eight treatments. The different letters indicate significant differences (ANOVA and EMMs post hoc; α = 0.05) between groups at the same week after priming.

In regard to the accumulation of TPC and FCs, treatments triggered a similar physiological reaction (Table A6 and Table A7). In trend, young and mature leaves accumulated higher TPC and FCs in thermoprimed, twice-stressed plants compared to non-primed plants, indicating a cross-tolerance that was triggered by thermopriming (Figure 2). Besides, young leaves of primed and stressed plants also showed higher FCs, selective for catechin, compared to non-primed and stressed plants. The opposite effect was shown in young leaves for decreased TPC and FC, selective for quercetin, after the subsequent salt treatments. Groups did not differ immediately after thermopriming, but they reacted differently to the recurrent stress treatments. Though, two months after the second stress, no differences were found in primed, non-primed, stressed, and non-stressed leaves of each age, respectively.

**Figure 3:**
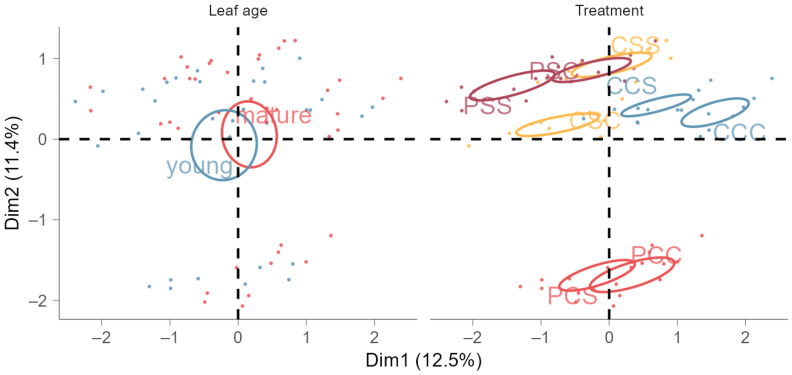
Multiple factor analysis of standardized primary and secondary leaf compounds for group means (specified by treatment, leaf age, and date; displayed as points for two leaf ages (young and mature) and the eight treatments as confidence ellipses with β = 0.95). Treatments: P represents thermopriming, C control conditions (non-primed or non-stressed groups), and S salt stress (e.g., C–C–C: control conditions (non-primed) instead of priming conditions, non-stressed (C) at the time of the first salt stress, and non-stressed (C) at the time of the second salt stress). Multiple factor analysis was performed on the active variable treatment as well as the supplementary variables: leaf age and date.

According to MFA, TCC, particularly chlorophyll a, and TAC explained most of the variability from all input variables (Figure A2b). These variables were positively correlated but negatively correlated with phenols and flavonoids. Additionally, non-primed treatments were clustered next to each other (Figure 3). In comparison, both the primed and non-stressed treatments, as well as the primed and once salt-stressed treatments, displayed overlapping clustering in the opposite quadrant to the control group. Moreover, the other primed and once- or twice-stressed treatments did also cluster apart from the control but showed a strong similarity to the non-primed, twice-stressed group. Although primed and non-primed plants showed a different physiological reaction in response to stress, the non-primed and subsequently stressed plants did not differ strongly from other non-primed groups, whereas thermoprimed plants showed opposite reactions in interaction with the subsequent salt stress events. Leaf age did not have a relevant effect due to an overlapping clustering of young and mature leaves.

For leaf pigments, thermoprimed and especially twice-stressed plants displayed a lower TCC in leaves in comparison to non-primed plants. The TCarC decreased in salt-stressed plants, whereas non-stressed thermoprimed and non-primed plants responded similarly to recurrent stress. Mature leaves of primed plants had a decreased TAC after thermopriming, though the subsequent stress resulted in a decrease of TAC in all treatments, with the most significant decrease observed in the primed ones. In contrast, TAC in young leaves was initially not affected by thermopriming or the first salt stress. Furthermore, thermopriming as well as subsequent stress events resulted in an accumulation of TPC and FCs in comparison to non-primed plants, which indicates a cross-tolerance to salt that was caused by thermopriming. These findings were also supported by MFA due to a separate clustering of thermoprimed and subsequently stressed plants compared to non-primed treatments that were generally clustered next to one another. As demonstrated for secondary metabolites in leaves, thermoprimed and non-primed plants showed a different physiological reaction in response to subsequent stresses. Therefore, thermopriming prepared plants to cope better with later stress due to an increased plant tolerance.

## 4. Discussion

Thermopriming of transplants is a novel approach to increasing plant tolerance against subsequent (a)biotic stress events [22,33]. For that reason, the effect of thermopriming and its interaction with subsequent, and particularly recurrent, salt stresses were evaluated in this study. Although many studies focus on single stresses (e.g., heat and salinity), only a few have addressed combined effects on plant growth, yield and fruit quality [7,8,9,10]. For that reason, this study evaluated the effects of two different types of abiotic stress, which were applied staggered as priming stimuli and two recurrent stress applications. Following, thermopriming and salinity will be discussed separately. Afterwards, their combination will be discussed to distinguish between single and combined effects on plant physiology and secondary plant metabolism.

Thermopriming led to significant delays in plant growth and development, similarly to results from other studies [18,34,35,36], while it increased plant tolerance through the accumulation of flavonols in leaves to cope with abiotic stress. These results are in accordance with a previous study [11]. We did not confirm accelerated plant growth at the same plant age as shown by Körner et al. [11], perhaps due to seasonal differences between both studies. Nevertheless, our results showed accelerated flowering in a later developmental stage when primed plants eventually caught up to the non-primed control in overall fruit yield [37]. Furthermore, initial growth deficits due to thermopriming were balanced out in a later plant development stage, similar to another study [35]. Although the general influence of heat on fruits and yield was studied before [22,38], yield performance and fruit quality of tomato plants after a controlled thermopriming of transplants were firstly presented here. We showed that the obtained (thermo-)memory by thermopriming resulted in an increased plant tolerance against stress through the accumulation of phenolic compounds in leaves [13]. Therefore, our findings indicated the well-known protective function of antioxidants such as phenols and flavonoids against reactive oxygen species that are generated under environmental stress [15,39]. Thus, the established thermomemory helped tomato plants adapt and respond stronger to subsequent stresses (thermotolerance). In this regard, thermomemory can be understood as a storage of molecular information that will be active once plants are exposed to stress again [19]. However, due to our experimental setup, we were not able to find a more rapid stress response to salinity in thermoprimed plants compared to control plants. Therefore, more frequent measurements of plant metabolites are needed.

Salt stress can lead to substantial changes in plant physiology and architecture [40]. In accordance with other studies [2,3,40,41,42,43,44,45], we observed a reduction in plant growth characterized by decreased plant height and number of leaves but not by fresh matter as a response to salt stress. We also found a decline in stomatal conductance in salt-stressed plants, which is associated with reduced plant growth [40], contrary to findings by Karaca et al. [3]. In contrast to Maggio et al. [40], none of our treatments resulted in decreased dry matter. Under salt stress, plants potentially exhibit a reduced photosynthesis rate, but tomato plants can tolerate salt stress to a certain extent without necessarily decreasing in growth and fruiting [2,46]. Similar to other studies on tomatoes, we found a reduced TCC in leaves [47,48], whereas TPC and FCs of salt-stressed plants increased to cope with the oxidative stress [48,49]. In contrast to Borghesi et al. [50], plants displayed decreased TAC and TCarC in leaves after the first and second salt stress.

The effect of salt stress on the number of fruits per infructescence was not evaluated in this study because each truss was limited to a maximum of six fruits, following the standard practice recommended by the cultivar’s breeder. Otherwise, the effect of salinity on yield may have been more pronounced in our experiment in contrast to other studies that observed a decrease in flowers and fruits [2,42]. In the first weeks of harvest, we found a reduction in fruit yield as expected in response to salt stress [2,3,40,42,43,51,52]. Though, in accordance with Mizrahi et al. [53], the overall yield did not differ from the control at a later stage of harvest anymore.

It is known that tomatoes grown under salinity can produce better fruit quality with increased sugars and acidity [7,42,51,53,54,55,56,57]. However, our findings did not indicate any effects of salt stress on the accumulation of glucose, fructose, or titratable acidity. Under non-stress growth conditions, glucose and fructose concentrations increase continuously during fruit ripening, whereas starch initially accumulates but eventually drastically decreases in ripe fruits [2]. In contrast to Mitchell et al. [54], we found increased starch concentrations in late infructescences at maturity. Moreover, we observed higher TCarC that accumulated as protective antioxidants under stress but no increased contents of ASC or FCs in fruits, in contrast to other studies, which can be explained by seasonal effects, the tomato cultivar, and the combination of stresses, respectively [7,58,59,60]. Also, Botella et al. [7] stated a predominant effect of heat on the ASC content in fruits.

The combination of salt and heat stress causes an even more severe reduction in plant biomass and yield compared to single stresses [7]. Salinity has reportedly a predominant effect on plant growth reduction compared to heat, which may make it more suitable for a subsequent stress treatment [8,9]. Furthermore, heat can improve the salinity tolerance of tomato plants [9]. In combination, these stresses led to a decrease in photosynthetic pigments [10]. Regarding fruits, we solely found effects on TCarC in fruits, although combined salt and heat can have an additive effect, causing an increase of phenolic compounds as well as an increase of glucose and fructose contents in fruits [7,8]. Botella et al. [7] summarized conflicting findings from several studies on the accumulation of phenolic compounds in fruits. We did not find any effects of thermopriming in combination with subsequent salt stress on TPC in fruits, which can be due to a non-selective determination of total contents that may have led to an underestimation [7]. In regard to the starch content in fruits, thermoprimed and once salt-stressed plants displayed a higher starch concentration in their fruits compared to non-primed and once salt-stressed plants, indicating a delayed fruit ripening process. However, this reason is unlikely because we did not find any effect of fruit coloration on the starch concentration between the treatments and harvested fruits per truss. Furthermore, fruit yield as well as the synthesis and accumulation of secondary compounds in fruits underlie external factors (e.g., season and day time) [61,62]. However, we did not find varying effects on fruit compounds depending on the harvest stage, except in TcarC, which was only accumulated in early trusses.

The single effects of heat and salt stress differ from those of combined and staggered stress, which are not yet well understood. We investigated thermopriming combined with subsequent salt stresses after periods of recovery, in contrast to other studies that mainly focused on the effects of one single stress or two simultaneously applied stresses. Thereby, we extended our understanding of the stress response in tomatoes concerning plant growth, yield, and the accumulation of primary and secondary metabolites in fruits and leaves for protection against abiotic stresses. In accordance with other studies on tomato plants, we showed a differential accumulation of various secondary metabolites in leaves and fruits that does not only protect plant tissues against oxidative stress caused by abiotic stress but also increases fruit quality [7,8]. Beyond that, we observed a physiological cross-talk between treatments in their secondary metabolites (TPC and FCs), which indicates a cross-tolerance to salinity triggered by thermopriming. Therefore, the induced cross-tolerance was a result of acquired thermotolerance [22]. We showed that the thermopriming of tomato transplants successfully prepared them for subsequent salt stress(es). This is relevant for producers because transplants are more susceptible to stress, such as salinity, compared to mature plants. Plant production under climate change can be secured when plants are primed for unpredictable stress events. However, the stresses in our study only occurred in the early developmental phase of tomato plants and had no effect on the overall fruit yield or quality. On the one hand, this can be considered a positive effect of thermopriming, but on the other hand, these findings also indicate that fruit yield is only affected by higher stress intensity and frequency. Thus, it has to be assessed whether thermopriming can mitigate continuous stress conditions after planting transplants in the greenhouse. Moreover, it needs to be evaluated whether a (thermo-)memory induced by thermopriming is still maintained after an extended recovery period without a stress trigger to effectively protect plants against subsequent stress(es) during fruit production in greenhouses. Then, thermopriming can be used as a sustainable method of crop protection to prepare plants against unpredictable future stress events and thus secure a stable fruit yield.

## Figures and Tables

**Table 1:** Summary of experimental settings.

Duration:	20 weeks
Period:	February–July 2023
Timing of thermopriming (week after sowing):	2nd
Timing of 1st salt stress (weeks after sowing):	3
Timing of 2nd salt stress (weeks after sowing):	5
Number of treatments:	8
Total number of blocks (incl. border):	6
Number of blocks with experimental plants:	4
Total number of plants (incl. border) per block:	8
Number of experimental plants per block:	6
Total number of experimental plants per treatment:	24
Number of harvested trusses per experimental plant:	3 (3rd/5th/7th truss per plant)

**Table 2:** Timeline with experimental treatments.

Treatment	Weeks after Sowing																					
	1	2	3		4	5		6	7	8	9	10	11	12	13	14	15	16	17	18	19	20
C–C–C																						
C–C–S																						
C–S–C																						
C–S–S																						
P–C–C																						
P–C–S																						
P–S–C																						
P–S–S																						

Notes: Seeding and 7-day lasting thermopriming (light red) in climate chambers, two single salt stress applications after WAS 3 and WAS 5 (blue) during greenhouse cultivation, six dates for leaf sampling (dark red vertical lines), and 5-week long duration of fruit sampling (dark grey) specific for each of the eight treatments over the whole experimental duration in weeks after sowing. Treatments: P represents thermopriming, C control conditions (non-primed or non-stressed groups), and S salt stress (e.g., C–C–C: control conditions (non-primed) instead of priming conditions, non-stressed (C) at the time of the first salt stress, and non-stressed (C) at the time of the second salt stress).

## Data Availability

The data presented in this study are available on request from the corresponding author due to costs for data publication.

## Appendix A

**Table A1:** Number of leaves, inflorescences and infructescences displayed as mean and standard deviation weeks after priming (WAP) per tomato plant differentiated for the eight treatments.

Treatment	WAP	Number of Leaves	n	Number of Inflorescences	n	Number of Infructescences	n
C–C–C	0	0.5 ± 0.5 ^b^	154				
	2	4.0 ± 0.4 ^b^	63				
	3	6.5 ± 0.6 ^b^	63	1.0 ± 0.0 ^a^	54		
	4	9.4 ± 0.5 ^c^	24	1.9 ± 0.3 ^c^	24		
	6	15.9 ± 0.5 ^c^	24	4.0 ± 0.0 ^c^	24		
	7	19.5 ± 0.8 ^c^	24	3.6 ± 0.5 ^a^	24	1.3 ± 0.5 ^ab^	24
	8	22.1 ± 1.1 ^b^	24	3.0 ± 0.5 ^a^	24	3.0 ± 0.3 ^b^	24
	10	27.8 ± 1.4 ^abc^	24	3.0 ± 0.8 ^a^	24	4.9 ± 0.4 ^b^	24
	12	33.8 ± 2.5 ^a^	24	2.1 ± 0.8 ^a^	24	6.9 ± 2.2 ^a^	24
C–C–S	4	8.9 ± 0.6 ^bc^	24	1.8 ± 0.4 ^bc^	24		
	6	15.2 ± 0.7 ^bc^	24	3.8 ± 0.4 ^bc^	24		
	7	19.2 ± 1.0 ^bc^	24	3.4 ± 0.5 ^a^	24	1.4 ± 0.5 ^ab^	23
	8	22.2 ± 1.6 ^b^	24	3.1 ± 0.4 ^a^	24	2.8 ± 0.4 ^ab^	24
	10	28.0 ± 1.5 ^abc^	23	3.1 ± 0.3 ^a^	23	4.8 ± 0.4 ^b^	23
	12	34.1 ± 1.6 ^a^	23	2.3 ± 0.5 ^a^	23	7.4 ± 0.8 ^a^	24
C–S–C	2	3.8 ± 0.4 ^a^	62				
	3	6.3 ± 0.7 ^ab^	62	1.0 ± 0.0 ^a^	46		
	4	9.0 ± 0.7 ^bc^	24	1.8 ± 0.4 ^bc^	24		
	6	15.5 ± 0.7 ^bc^	24	3.8 ± 0.4 ^bc^	24		
	7	19.2 ± 0.9 ^bc^	24	3.3 ± 0.6 ^a^	24	1.5 ± 0.6 ^b^	24
	8	22.2 ± 1.0 ^b^	24	3.0 ± 0.3 ^a^	24	2.8 ± 0.4 ^ab^	24
	10	28.1 ± 1.0 ^bc^	24	3.2 ± 0.4 ^ab^	24	4.9 ± 0.4 ^b^	24
	12	34.2 ± 1.0 ^a^	24	2.2 ± 0.5 ^a^	24	7.4 ± 0.6 ^a^	24
C–S–S	4	8.6 ± 0.6 ^ab^	24	1.6 ± 0.5 ^abc^	24		
	6	14.9 ± 0.9 ^ab^	24	3.8 ± 0.4 ^bc^	24		
	7	18.5 ± 1.0 ^ab^	24	3.4 ± 0.5 ^a^	24	1.3 ± 0.5 ^ab^	23
	8	21.5 ± 1.1 ^b^	24	2.9 ± 0.5 ^a^	24	2.8 ± 0.4 ^ab^	24
	10	27.2 ± 1.3 ^ab^	24	3.0 ± 0.2 ^a^	24	4.7 ± 0.5 ^ab^	24
	12	33.5 ± 1.5 ^a^	24	2.2 ± 0.6 ^a^	24	7.1 ± 0.4 ^a^	24
P–C–C	0	0.0 ± 0.2 ^a^	154				
	2	4.0 ± 0.4 ^ab^	63				
	3	6.5 ± 0.6 ^b^	63	1.0 ± 0.0 ^a^	51		
	4	9.1 ± 0.5 ^bc^	24	1.7 ± 0.5 ^bc^	24		
	6	15.8 ± 0.8 ^c^	24	3.7 ± 0.6 ^bc^	24		
	7	19.3 ± 0.8 ^bc^	24	3.7 ± 0.5 ^a^	24	1.2 ± 0.4 ^ab^	22
	8	22.3 ± 0.8 ^b^	24	3.2 ± 0.6 ^a^	24	2.9 ± 0.7 ^ab^	24
	10	28.4 ± 0.8 ^c^	24	3.2 ± 0.4 ^ab^	24	4.7 ± 0.7 ^ab^	24
	12	34.1 ± 1.1 ^a^	24	2.2 ± 0.4 ^a^	24	7.2 ± 0.7 ^a^	24
P–C–S	4	9.0 ± 0.6 ^bc^	24	1.5 ± 0.5 ^abc^	24		
	6	15.2 ± 0.8 ^bc^	24	3.6 ± 0.5 ^ab^	24		
	7	18.8 ± 1.0 ^bc^	24	3.5 ± 0.5 ^a^	24	1.2 ± 0.4 ^ab^	24
	8	21.5 ± 1.3 ^b^	24	3.0 ± 0.5 ^a^	24	2.7 ± 0.5 ^ab^	24
	10	27.4 ± 1.3 ^abc^	24	3.2 ± 0.4 ^ab^	24	4.7 ± 0.5 ^ab^	24
	12	33.3 ± 1.8 ^a^	24	2.4 ± 0.5 ^a^	24	7.3 ± 0.6 ^a^	24
P–S–C	2	3.8 ± 0.5 ^a^	63				
	3	6.2 ± 0.7 ^a^	63	1.0 ± 0.0 ^a^	36		
	4	8.9 ± 0.6 ^ab^	24	1.5 ± 0.5 ^ab^	24		
	6	15.4 ± 0.7 ^bc^	24	3.7 ± 0.5 ^abc^	24		
	7	19.0 ± 1.0 ^bc^	24	3.5 ± 0.6 ^a^	24	1.3 ± 0.5 ^ab^	24
	8	21.6 ± 1.1 ^b^	24	2.9 ± 0.4 ^a^	24	2.9 ± 0.3 ^ab^	24
	10	27.6 ± 1.2 ^abc^	23	3.3 ± 0.5 ^ab^	23	4.6 ± 0.5 ^ab^	23
	12	33.3 ± 1.1 ^a^	23	2.2 ± 0.7 ^a^	24	7.0 ± 1.5 ^a^	24
P–S–S	4	8.4 ± 0.7 ^a^	24	1.2 ± 0.4 ^a^	24		
	6	14.4 ± 1.2 ^a^	24	3.3 ± 0.5 ^a^	24		
	7	18.0 ± 1.1 ^a^	24	3.4 ± 0.5 ^a^	24	1.1 ± 0.3 ^a^	22
	8	20.4 ± 1.0 ^a^	24	3.1 ± 0.4 ^a^	24	2.6 ± 0.5 ^a^	24
	10	26.9 ± 1.4 ^a^	24	3.5 ± 0.7 ^b^	24	4.3 ± 0.7 ^a^	24
	12	33.0 ± 1.6 ^a^	24	2.4 ± 0.5 ^a^	24	7.0 ± 0.5 ^a^	24

Notes: treatments: P represents thermopriming, C control conditions (non-primed or non-stressed groups), and S salt stress (e.g., C–C–C: control conditions (non-primed) instead of priming conditions, non-stressed (C) at the time of the first salt stress, and non-stressed (C) at the time of the second salt stress). The different letters indicate significant differences (ANOVA and EMMs post hoc; α = 0.05) between groups in the same WAP of the corresponding parameter. n corresponds to the sample size of the previous parameters.

**Table A2:** Fresh matter (FM), dry matter (DM), and accumulated fruit yield displayed as mean and standard deviation at the end of the experiment of tomato plants differentiated for the eight treatments.

Treatment	FM	DM	n	Fruit Yield	n
	[g]			[g]	
C–C–C	2321 ± 293 ^a^	316 ± 61 ^a^	15	4682 ± 836 ^a^	24
C–C–S	2421 ± 434 ^a^	316 ± 58 ^a^	15	4572 ± 581 ^a^	24
C–S–C	2485 ± 235 ^a^	338 ± 37 ^a^	15	4722 ± 596 ^a^	24
C–S–S	2191 ± 289 ^a^	299 ± 46 ^a^	15	4881 ± 693 ^a^	24
P–C–C	2513 ± 273 ^a^	341 ± 51 ^a^	15	4786 ± 497 ^a^	24
P–C–S	2207 ± 306 ^a^	294 ± 42 ^a^	15	4772 ± 548 ^a^	24
P–S–C	2284 ± 348 ^a^	305 ± 51 ^a^	15	4809 ± 658 ^a^	23
P–S–S	2220 ± 274 ^a^	302 ± 49 ^a^	15	4699 ± 587 ^a^	24

Notes: treatments: P represents thermopriming, C control conditions (non-primed or non-stressed groups), and S salt stress (e.g., C–C–C: control conditions (non-primed) instead of priming conditions, non-stressed (C) at the time of the first salt stress, and non-stressed (C) at the time of the second salt stress). The different letters indicate significant differences (ANOVA and EMMs post hoc; α = 0.05) between treatments of the corresponding parameter. n corresponds to the sample size of the previous parameters.

**Table A3:** Stomatal conductance of water vapor (g_sw_) and electron transport rate (ETR) displayed as mean and standard deviation weeks after priming (WAP) per tomato plant differentiated for the eight treatments.

Treatment	WAP	g_sw_	ETR	n
		[mol m^−2^ s^−1^]	[μmol e^−^ m^−2^ s^−1^]	
C–C–C	3 ^1^	1.01 ± 0.14 ^c^	52.21 ± 11.06 ^b^	63
	3 ^2^	0.94 ± 0.12 ^e^	23.17 ± 5.88 ^a^	32
	4	0.80 ± 0.20 ^cd^	54.15 ± 20.41 ^a^	24
	7	0.75 ± 0.16 ^a^	35.84 ± 12.96 ^ab^	24
	9	1.23 ± 0.43 ^a^	125.02 ± 33.21 ^a^	21
	11	1.00 ± 0.21 ^ab^	72.79 ± 28.55 ^ab^	24
	13	0.36 ± 0.19 ^ab^	49.67 ± 62.32 ^a^	24
C–C–S	3	0.81 ± 0.18 ^cd^	27.05 ± 5.71 ^abc^	30
	4	0.60 ± 0.22 ^b^	68.98 ± 43.09 ^ab^	24
	7	0.72 ± 0.09 ^a^	31.53 ± 7.29 ^a^	24
	9	1.29 ± 0.33 ^a^	118.42 ± 56.78 ^a^	21
	11	1.10 ± 0.26 ^b^	76.24 ± 36.98 ^ab^	24
	13	0.41 ± 0.14 ^ab^	44.29 ± 46.15 ^a^	24
C–S–C	3	0.88 ± 0.17 ^ab^	51.77 ± 12.46 ^b^	62
	3	0.71 ± 0.16 ^bc^	28.52 ± 8.94 ^bc^	32
	4	0.69 ± 0.21 ^bc^	75.70 ± 41.46 ^ab^	24
	7	0.80 ± 0.10 ^a^	37.52 ± 17.14 ^ab^	24
	9	1.23 ± 0.30 ^a^	163.30 ± 67.37 ^a^	21
	11	1.04 ± 0.28 ^ab^	54.88 ± 18.46 ^a^	24
	13	0.32 ± 0.13 ^ab^	53.30 ± 59.30 ^a^	24
C–S–S	3	0.41 ± 0.22 ^a^	30.57 ± 10.78 ^c^	32
	4	0.34 ± 0.17 ^a^	83.65 ± 46.83 ^b^	24
	7	0.78 ± 0.14 ^a^	43.55 ± 25.10 ^b^	24
	9	1.25 ± 0.20 ^a^	158.40 ± 59.59 ^a^	19
	11	1.07 ± 0.17 ^ab^	81.95 ± 36.76 ^b^	24
	13	0.36 ± 0.10 ^ab^	62.58 ± 74.03 ^a^	24
P–C–C	3	0.94 ± 0.14 ^bc^	46.67 ± 6.82 ^a^	63
	3	0.84 ± 0.09 ^de^	31.03 ± 13.90 ^c^	30
	4	0.92 ± 0.24 ^d^	53.05 ± 22.56 ^a^	24
	7	0.70 ± 0.12 ^a^	32.53 ± 3.74 ^a^	24
	9	1.18 ± 0.26 ^a^	137.14 ± 59.63 ^a^	22
	11	0.89 ± 0.32 ^a^	68.52 ± 34.48 ^ab^	24
	13	0.31 ± 0.15 ^a^	54.28 ± 49.49 ^a^	24
P–C–S	3	0.68 ± 0.16 ^b^	25.57 ± 15.11 ^ab^	32
	4	0.65 ± 0.15 ^bc^	55.11 ± 20.52 ^a^	24
	7	0.76 ± 0.20 ^a^	38.48 ± 11.20 ^ab^	24
	9	1.05 ± 0.21 ^a^	141.52 ± 63.80 ^a^	20
	11	1.04 ± 0.19 ^ab^	72.64 ± 30.84 ^ab^	24
	13	0.43 ± 0.10 ^b^	63.47 ± 67.12 ^a^	24
P–S–C	3	0.83 ± 0.14 ^a^	51.34 ± 11.56 ^b^	63
	3	0.70 ± 0.13 ^bc^	25.35 ± 5.25 ^ab^	32
	4	0.55 ± 0.17 ^b^	70.69 ± 44.22 ^ab^	24
	7	0.77 ± 0.12 ^a^	38.94 ± 12.00 ^ab^	24
	9	1.20 ± 0.22 ^a^	120.58 ± 53.35 ^a^	18
	11	0.93 ± 0.29 ^ab^	75.64 ± 28.40 ^ab^	23
	13	0.36 ± 0.16 ^ab^	48.83 ± 55.93 ^a^	24
P–S–S	3	0.41 ± 0.23 ^a^	22.98 ± 10.53 ^a^	31
	4	0.36 ± 0.20 ^a^	79.40 ± 41.42 ^ab^	24
	7	0.79 ± 0.17 ^a^	39.30 ± 8.60 ^ab^	24
	9	1.11 ± 0.18 ^a^	131.10 ± 58.95 ^a^	22
	11	1.07 ± 0.16 ^ab^	70.06 ± 18.76 ^ab^	24
	13	0.40 ± 0.13 ^ab^	61.94 ± 61.02 ^a^	24

^1^ First measurement just before the second salt stress application (same day); ^2^ second measurement three days after the second salt stress application. Notes: treatments: P represents thermopriming, C control conditions (non-primed or non-stressed groups), and S salt stress (e.g., C–C–C: control conditions (non-primed) instead of priming conditions, non-stressed (C) at the time of the first salt stress, and non-stressed (C) at the time of the second salt stress). The different letters indicate significant differences (ANOVA and EMMs post hoc; α = 0.05) between groups in the same WAP of the corresponding parameter. n corresponds to the sample size of the previous parameters.

**Table A4:** Fruit compounds displayed as mean and standard deviation in early, intermediate, and late trusses per tomato plant differentiated for the eight treatments.

Treatment	Stage of Harvest	TCarC ^1^	TAC ^2^	TPC ^3^	FC_Catechin_ ^4^	FC_Quercetin_ ^4^	n
		[µg mg^−1^ DM^−1^]	[µg CyEs mg^−1^ DM^−1^]	[µg GAEs mg^−1^ DM^−1^]	[µg CEs mg^−1^ DM^−1^]	[µg QEs mg^−1^ DM^−1^]	
C–C–C	early ^5^	1.0 ± 0.2 ^ab^	0.7 ± 0.3 ^a^	3.3 ± 0.2 ^a^	4.2 ± 0.8 ^a^	3.1 ± 0.4 ^a^	9
C–C–S		1.0 ± 0.2 ^ab^	0.9 ± 0.4 ^a^	3.7 ± 0.5 ^a^	4.6 ± 1.0 ^a^	3.1 ± 0.7 ^a^	9
C–S–C		1.0 ± 0.3 ^ab^	0.9 ± 0.3 ^a^	3.6 ± 0.8 ^a^	4.5 ± 1.0 ^a^	3.3 ± 0.4 ^a^	10
C–S–S		0.8 ± 0.2 ^a^	1.0 ± 0.3 ^a^	3.5 ± 0.2 ^a^	4.9 ± 1.0 ^a^	3.3 ± 0.3 ^a^	7
P–C–C		1.0 ± 0.2 ^ab^	0.9 ± 0.5 ^a^	3.6 ± 0.3 ^a^	4.7 ± 1.4 ^a^	3.3 ± 0.5 ^a^	10
P–C–S		1.0 ± 0.3 ^ab^	0.7 ± 0.1 ^a^	3.5 ± 0.1 ^a^	4.0 ± 0.6 ^a^	3.2 ± 0.4 ^a^	10
P–S–C		1.0 ± 0.1 ^ab^	0.7 ± 0.4 ^a^	3.4 ± 0.2 ^a^	3.9 ± 1.1 ^a^	3.1 ± 0.5 ^a^	9
P–S–S		1.1 ± 0.1 ^b^	0.9 ± 0.3 ^a^	3.5 ± 0.3 ^a^	4.5 ± 0.8 ^a^	3.4 ± 0.6 ^a^	11
C–C–C	inter- mediate ^6^	1.0 ± 0.3 ^a^	0.9 ± 0.3 ^ab^	3.9 ± 0.2 ^a^	4.0 ± 0.8 ^ab^	3.3 ± 0.4 ^a^	10
C–C–S		1.0 ± 0.3 ^a^	0.8 ± 0.3 ^a^	3.8 ± 0.3 ^a^	3.7 ± 0.6 ^a^	3.1 ± 0.5 ^a^	12
C–S–C		0.9 ± 0.2 ^a^	1.2 ± 0.2 ^b^	3.9 ± 0.3 ^a^	5.0 ± 1.0 ^b^	3.6 ± 0.3 ^a^	10
C–S–S		1.0 ± 0.3 ^a^	1.1 ± 0.3 ^ab^	3.9 ± 0.4 ^a^	4.7 ± 0.9 ^ab^	3.5 ± 0.3 ^a^	12
P–C–C		1.0 ± 0.3 ^a^	0.7 ± 0.4 ^a^	3.9 ± 0.4 ^a^	4.1 ± 1.5 ^ab^	3.3 ± 0.6 ^a^	9
P–C–S		0.8 ± 0.2 ^a^	0.9 ± 0.3 ^ab^	3.8 ± 0.4 ^a^	4.3 ± 0.9 ^ab^	3.4 ± 0.4 ^a^	10
P–S–C		0.9 ± 0.2 ^a^	0.8 ± 0.3 ^ab^	3.8 ± 0.3 ^a^	4.2 ± 1.1 ^ab^	3.1 ± 0.5 ^a^	10
P–S–S		0.9 ± 0.2 ^a^	1.0 ± 0.5^ab^	4.1 ± 0.3 ^a^	4.8 ± 1.3 ^ab^	3.4 ± 0.4 ^a^	11
C–C–C	late ^7^	1.1 ± 0.2 ^a^	0.9 ± 0.4 ^a^	4.5 ± 0.3 ^a^	4.4 ± 0.8 ^a^	3.6 ± 0.5 ^a^	10
C–C–S		1.2 ± 0.1 ^a^	0.9 ± 0.4 ^a^	4.9 ± 0.3 ^a^	5.0 ± 1.8 ^a^	3.6 ± 0.5 ^a^	10
C–S–C		1.1 ± 0.2 ^a^	0.8 ± 0.3 ^a^	4.8 ± 0.2 ^a^	4.4 ± 1.0 ^a^	3.6 ± 0.6 ^a^	11
C–S–S		1.2 ± 0.2 ^a^	1.0 ± 0.3 ^a^	5.0 ± 0.6 ^a^	5.3 ± 1.3 ^a^	3.8 ± 0.5 ^a^	11
P–C–C		1.1 ± 0.2 ^a^	1.0 ± 0.5 ^a^	4.9 ± 0.4 ^a^	5.2 ± 2.3 ^a^	3.7 ± 0.4 ^a^	10
P–C–S		1.2 ± 0.2 ^a^	1.0 ± 0.2 ^a^	5.0 ± 0.4 ^a^	5.2 ± 1.0 ^a^	3.6 ± 0.3 ^a^	12
P–S–C		1.1 ± 0.2 ^a^	1.1 ± 0.5 ^a^	4.8 ± 0.4 ^a^	5.2 ± 2.0 ^a^	3.6 ± 0.7 ^a^	11
P–S–S		1.1 ± 0.2 ^a^	1.0 ± 0.5 ^a^	4.9 ± 0.4 ^a^	5.9 ± 1.9 ^a^	3.7 ± 0.7 ^a^	11

^1^ TCarC: total carotenoid content; ^2^ TAC: total anthocyanin content (expressed as CyEs, cyanidin-3,5-O-diglucosid equivalents); ^3^ TPC: total phenolic content (expressed as GAEs, gallic acid equivalents); ^4^ FC: flavonoid content (expressed as CEs, catechin equivalents, or QEs, quercetin equivalents); ^5^ early: 3rd truss per plant; ^6^ intermediate: 5th truss per plant; ^7^ late: 7th truss per plant. Notes: treatments: P represents thermopriming, C control conditions (non-primed or non-stressed groups), and S salt stress (e.g., C–C–C: control conditions (non-primed) instead of priming conditions, non-stressed (C) at the time of the first salt stress, and non-stressed (C) at the time of the second salt stress). The different letters indicate significant differences (ANOVA and EMMs post hoc; α = 0.05) between groups with the same stage of harvest of the corresponding parameter. n corresponds to the sample size of the previous parameters.

**Table A5:** Fruit compounds displayed as mean and standard deviation in early, intermediate, and late trusses per tomato plant differentiated for the eight treatments.

Treatment	Stage of Harvest	ASC ^1^	n	Glucose	Fructose	Starch	n	TA ^2^	n
		[mg kg^−1^ fruit FM^−1^]		[µg mg^−1^ fruit FM^−1^]	[µg mg^−1^ fruit FM^−1^]	[µg mg^−1^ fruit FM^−1^]		[µg CAEs mg^−1^ DM^−1^]	
C–C–C	early ^3^	231.5 ± 19.7 ^ab^	12	220.8 ± 42.4 ^bc^	219.3 ± 34.6 ^ab^	4.4 ± 1.0 ^a^	9	2.9 ± 0.4 ^a^	11
C–C–S		226.0 ± 15.3 ^ab^	12	217.6 ± 32.4 ^bc^	211.7 ± 22.0 ^ab^	4.7 ± 1.8 ^a^	10	2.8 ± 0.4 ^a^	9
C–S–C		236.7 ± 37.1 ^b^	12	239.0 ± 34.8 ^c^	238.0 ± 31.0 ^b^	4.7 ± 1.7 ^a^	11	2.9 ± 0.3 ^a^	10
C–S–S		214.8 ± 15.6 ^ab^	12	192.2 ± 33.0 ^ab^	199.4 ± 30.9 ^ab^	4.4 ± 1.2 ^a^	12	2.8 ± 0.3 ^a^	12
P–C–C		236.8 ± 20.2 ^b^	11	213.0 ± 28.1 ^bc^	214.6 ± 21.7 ^ab^	4.4 ± 1.6 ^a^	12	2.9 ± 0.4 ^a^	12
P–C–S		207.8 ± 12.0 ^a^	11	194.3 ± 40.3 ^ab^	203.6 ± 35.4 ^ab^	3.9 ± 1.7 ^a^	12	2.6 ± 0.5 ^a^	12
P–S–C		212.5 ± 14.5 ^ab^	12	205.5 ± 28.2 ^abc^	215.3 ± 35.4 ^ab^	4.5 ± 1.8 ^a^	10	2.8 ± 0.4 ^a^	11
P–S–S		218.1 ± 11.9 ^ab^	11	165.7 ± 38.0 ^a^	178.8 ± 29.6 ^a^	4.8 ± 1.5 ^a^	12	3.0 ± 0.5 ^a^	11
C–C–C	inter- mediate ^4^	261.7 ± 34.9 ^a^	12	205.4 ± 27.4 ^ab^	211.9 ± 28.0 ^ab^	2.4 ± 2.8 ^a^	12	2.9 ± 0.3 ^a^	12
C–C–S		255.3 ± 23.1 ^a^	12	187.3 ± 24.6 ^ab^	189.9 ± 25.0 ^ab^	2.4 ± 1.3 ^a^	12	2.7 ± 0.2 ^a^	12
C–S–C		269.2 ± 19.6 ^a^	12	200.2 ± 25.4 ^ab^	206.2 ± 25.5 ^ab^	2.3 ± 1.0 ^a^	11	2.8 ± 0.5 ^a^	12
C–S–S		262.5 ± 34.4 ^a^	12	198.1 ± 32.4 ^ab^	204.7 ± 34.0 ^ab^	2.5 ± 1.2 ^a^	12	2.8 ± 0.2 ^a^	12
P–C–C		284.1 ± 38.7 ^a^	12	229.8 ± 42.7 ^b^	236.7 ± 48.4 ^b^	4.0 ± 2.5 ^a^	12	2.7 ± 0.5 ^a^	12
P–C–S		258.3 ± 27.9 ^a^	12	181.6 ± 23.0 ^a^	188.0 ± 20.4 ^a^	2.6 ± 2.4 ^a^	11	2.9 ± 0.2 ^a^	12
P–S–C		264.5 ± 29.1 ^a^	12	191.4 ± 28.2 ^a^	199.0 ± 26.9 ^a^	2.4 ± 1.5 ^a^	12	3.0 ± 0.4 ^a^	12
P–S–S		257.0 ± 19.2 ^a^	12	207.1 ± 43.6 ^ab^	217.2 ± 46.1 ^ab^	3.2 ± 2.5 ^a^	12	2.8 ± 0.4 ^a^	12
C–C–C	late ^5^	323.5 ± 28.5 ^a^	11	241.8 ± 60.7 ^a^	256.4 ± 55.0 ^a^	4.1 ± 3.0 ^ab^	11	3.0 ± 0.3 ^b^	11
C–C–S		331.9 ± 26.1 ^a^	12	249.6 ± 65.2 ^a^	273.0 ± 58.9 ^a^	2.7 ± 1.0 ^a^	12	3.0 ± 0.4 ^ab^	12
C–S–C		333.4 ± 21.2 ^a^	12	246.8 ± 55.1 ^a^	265.4 ± 54.0 ^a^	2.3 ± 1.0 ^a^	11	3.0 ± 0.3 ^ab^	12
C–S–S		324.8 ± 32.8 ^a^	12	214.0 ± 60.0 ^a^	240.6 ± 58.2 ^a^	3.4 ± 2.3 ^ab^	11	2.9 ± 0.2 ^ab^	12
P–C–C		335.9 ± 30.2 ^a^	12	228.0 ± 56.3 ^a^	244.1 ± 49.5 ^a^	4.4 ± 3.0 ^ab^	12	2.9 ± 0.4 ^ab^	12
P–C–S		310.2 ± 25.3 ^a^	12	199.6 ± 55.2 ^a^	223.0 ± 52.2 ^a^	6.3 ± 4.1 ^b^	12	2.8 ± 0.4 ^ab^	12
P–S–C		305.1 ± 32.1 ^a^	12	223.7 ± 59.3 ^a^	237.5 ± 55.6 ^a^	3.5 ± 2.3 ^ab^	12	3.0 ± 0.3 ^ab^	11
P–S–S		314.6 ± 30.4 ^a^	12	217.6 ± 71.3 ^a^	246.5 ± 64.4 ^a^	3.2 ± 1.7 ^ab^	12	2.7 ± 0.3 ^a^	12

^1^ ASC: ascorbic acid; ^2^ TA: titratable acidity (expressed as CAEs, citric acid equivalents); ^3^ early: 3rd truss per plant; ^4^ intermediate: 5th truss per plant; ^5^ late: 7th truss per plant. Notes: treatments: P represents thermopriming, C control conditions (non-primed or non-stressed groups), and S salt stress (e.g., C–C–C: control conditions (non-primed) instead of priming conditions, non-stressed (C) at the time of the first salt stress, and non-stressed (C) at the time of the second salt stress). The different letters indicate significant differences (ANOVA and EMMs post hoc; α = 0.05) between groups with the same stage of harvest of the corresponding parameter. n corresponds to the sample size of the previous parameters.

**Table A6:** Leaf compounds displayed as mean and standard deviation in young tomato leaves differentiated for the eight treatments weeks after priming (WAP).

Treatment	WAP	TCC ^1^	TCarC ^2^	TAC ^3^	TPC ^4^	FC_Catechin_ ^5^	FC_Quercetin_ ^5^	n
		[µg mg^−1^ DM^−1^]		[µg CyEs mg^−1^ DM^−1^]	[µg GAEs mg^−1^ DM^−1^]	[µg CEs mg^−1^ DM^−1^]	[µg QEs mg^−1^ DM^−1^]	
C–C–C	1	4.2 ± 0.2 ^b^	3.4 ± 0.3 ^b^	3.0 ± 0.4 ^a^	6.7 ± 0.6 ^a^	13.3 ± 2.8 ^a^	12.8 ± 0.4 ^a^	16
	3	4.7 ± 0.4 ^a^	4.2 ± 0.3 ^ab^	2.3 ± 0.5 ^a^	10.9 ± 2.5 ^b^	21.3 ± 4.6 ^a^	15.4 ± 1.1 ^ab^	8
	4	5.1 ± 0.5 ^b^	3.4 ± 0.6 ^c^	3.1 ± 0.8 ^ab^	11.0 ± 1.4 ^e^	24.7 ± 2.5 ^a^	15.0 ± 0.6 ^d^	12
	5	5.1 ± 0.3 ^a^	3.8 ± 0.6 ^a^	5.5 ± 0.7 ^bc^	10.5 ± 1.0 ^a^	28.0 ± 2.8 ^bc^	16.2 ± 1.5 ^a^	12
	9	3.8 ± 0.5 ^b^	2.6 ± 0.4 ^a^	4.5 ± 0.9 ^a^	17.1 ± 1.5 ^a^	41.9 ± 20.4 ^a^	18.0 ± 0.9 ^a^	12
	13	3.1 ± 0.3 ^c^	1.7 ± 0.2 ^a^	3.6 ± 0.5 ^b^	15.7 ± 1.2 ^a^	29.9 ± 5.0 ^a^	17.6 ± 2.2 ^a^	12
C–C–S	4	5.1 ± 0.4 ^b^	2.8 ± 0.7 ^bc^	3.2 ± 0.5 ^b^	10.0 ± 1.2 ^de^	24.2 ± 6.1 ^a^	14.4 ± 0.9 ^cd^	12
	5	5.2 ± 0.5 ^a^	3.8 ± 0.7 ^a^	4.0 ± 1.3 ^a^	10.5 ± 0.8 ^a^	22.8 ± 2.4 ^a^	16.8 ± 1.0 ^a^	12
	9	3.6 ± 0.4 ^ab^	2.6 ± 0.2 ^a^	4.7 ± 0.8 ^a^	16.6 ± 1.4 ^a^	42.1 ± 22.9 ^a^	17.5 ± 0.8 ^a^	12
	13	3.0 ± 0.4 ^bc^	1.7 ± 0.2 ^a^	3.5 ± 0.6 ^b^	16.0 ± 2.0 ^a^	29.1 ± 3.9 ^a^	17.3 ± 1.3 ^a^	12
C–S–C	3	4.6 ± 0.5 ^a^	3.4 ± 1.3 ^a^	2.3 ± 0.6 ^a^	9.5 ± 2.3 ^ab^	21.2 ± 2.9 ^a^	14.7 ± 0.8 ^ab^	8
	4	5.0 ± 0.5 ^ab^	2.8 ± 1.0 ^bc^	3.3 ± 0.4 ^b^	8.4 ± 1.4 ^abc^	19.9 ± 4.0 ^a^	12.6 ± 1.0 ^ab^	12
	5	4.7 ± 1.3 ^a^	3.8 ± 0.7 ^a^	4.1 ± 1.9 ^a^	9.5 ± 2.9 ^a^	22.2 ± 6.3 ^a^	15.1 ± 4.4 ^a^	12
	9	3.4 ± 0.4 ^ab^	2.5 ± 0.2 ^a^	4.1 ± 0.5 ^a^	17.2 ± 1.1 ^a^	42.8 ± 21.2 ^ab^	17.7 ± 0.7 ^a^	12
	13	2.5 ± 0.3 ^a^	1.4 ± 0.2 ^a^	2.7 ± 0.6 ^a^	17.4 ± 1.3 ^a^	33.0 ± 2.6 ^a^	16.9 ± 1.6 ^a^	12
C–S–S	4	4.7 ± 0.4 ^ab^	2.8 ± 0.7 ^bc^	3.2 ± 0.5 ^b^	8.3 ± 0.8 ^ab^	20.7 ± 6.0 ^a^	13.2 ± 1.8 ^ab^	12
	5	5.3 ± 0.3 ^a^	3.9 ± 0.7 ^a^	6.2 ± 0.7 ^c^	9.9 ± 1.2 ^a^	25.5 ± 1.6 ^abc^	15.8 ± 0.5 ^a^	12
	9	3.6 ± 0.5 ^ab^	2.5 ± 0.3 ^a^	4.0 ± 0.7 ^a^	17.5 ± 1.9 ^a^	42.8 ± 20.4 ^ab^	17.7 ± 1.0 ^a^	12
	13	2.9 ± 0.3 ^abc^	1.6 ± 0.2 ^a^	3.2 ± 0.7 ^ab^	15.9 ± 1.7 ^a^	31.0 ± 2.3 ^a^	17.1 ± 1.7 ^a^	12
P–C–C	1	4.0 ± 0.3 ^a^	3.1 ± 0.4 ^a^	3.1 ± 0.5 ^a^	7.0 ± 0.9 ^a^	12.6 ± 2.6 ^a^	13.0 ± 1.4 ^a^	16
	3	4.8 ± 0.3 ^a^	4.4 ± 0.2 ^b^	2.2 ± 0.5 ^a^	10.5 ± 1.0 ^ab^	23.8 ± 3.2 ^a^	15.7 ± 0.7 ^b^	8
	4	5.0 ± 0.4 ^b^	3.0 ± 0.4 ^bc^	2.7 ± 0.5 ^ab^	9.5 ± 1.0 ^bcd^	22.7 ± 4.2 ^a^	13.5 ± 0.7 ^abc^	12
	5	5.0 ± 0.5 ^a^	3.8 ± 0.7 ^a^	4.1 ± 1.7 ^a^	11.0 ± 0.9 ^a^	23.6 ± 2.5 ^ab^	17.0 ± 0.9 ^a^	12
	9	3.2 ± 0.4 ^a^	2.4 ± 0.2 ^a^	4.0 ± 0.7 ^a^	17.7 ± 1.3 ^a^	43.6 ± 17.1 ^ab^	17.3 ± 1.2 ^a^	12
	13	2.6 ± 0.3 ^ab^	1.6 ± 0.3 ^a^	3.0 ± 0.6 ^ab^	16.5 ± 1.7 ^a^	31.3 ± 7.2 ^a^	17.0 ± 1.7 ^a^	12
P–C–S	4	4.8 ± 0.3 ^ab^	2.4 ± 0.5 ^b^	2.7 ± 0.3 ^ab^	9.7 ± 1.3 ^cde^	23.9 ± 7.1 ^a^	12.9 ± 0.9 ^ab^	12
	5	5.0 ± 0.4 ^a^	3.8 ± 0.7 ^a^	4.2 ± 1.7 ^ab^	10.6 ± 1.3 ^a^	29.6 ± 3.7 ^c^	16.2 ± 1.2 ^a^	12
	9	3.5 ± 0.4 ^ab^	2.6 ± 0.3 ^a^	4.2 ± 0.6 ^a^	17.6 ± 0.9 ^a^	47.1 ± 21.1 ^b^	17.3 ± 1.0 ^a^	11
	13	2.9 ± 0.4 ^abc^	1.6 ± 0.3 ^a^	3.1 ± 0.7 ^ab^	16.2 ± 2.1 ^a^	33.9 ± 6.0 ^a^	16.8 ± 1.5 ^a^	12
P–S–C	3	4.6 ± 0.5 ^a^	3.6 ± 0.5 ^ab^	2.2 ± 0.6 ^a^	8.2 ± 1.1 ^a^	20.3 ± 3.5 ^a^	14.4 ± 0.7 ^a^	8
	4	4.9 ± 0.4 ^ab^	2.3 ± 0.4 ^b^	3.1 ± 0.5 ^ab^	7.7 ± 1.0 ^a^	19.9 ± 3.4 ^a^	13.5 ± 0.4 ^bc^	12
	5	5.0 ± 0.3 ^a^	3.9 ± 0.6 ^a^	5.6 ± 0.6 ^c^	10.7 ± 1.1 ^a^	28.6 ± 2.3 ^c^	16.1 ± 1.6 ^a^	12
	9	3.5 ± 0.3 ^ab^	2.5 ± 0.3 ^a^	4.2 ± 0.5 ^a^	17.9 ± 1.3 ^a^	45.7 ± 17.9 ^ab^	17.4 ± 1.0 ^a^	12
	13	2.8 ± 0.4 ^abc^	1.6 ± 0.3 ^a^	3.2 ± 0.5 ^ab^	16.3 ± 1.7 ^a^	33.0 ± 4.5 ^a^	17.0 ± 2.5 ^a^	12
P–S–S	4	4.4 ± 0.5 ^a^	0.8 ± 1.4 ^a^	2.5 ± 0.6 ^a^	7.7 ± 0.6 ^a^	22.8 ± 5.3 ^a^	12.4 ± 0.6 ^a^	12
	5	5.1 ± 0.4 ^a^	3.8 ± 0.7 ^a^	5.6 ± 0.5 ^c^	10.4 ± 1.0 ^a^	28.8 ± 5.5 ^c^	15.8 ± 0.7 ^a^	12
	9	3.5 ± 0.5 ^ab^	2.6 ± 0.4 ^a^	4.0 ± 0.6 ^a^	17.7 ± 1.7 ^a^	45.2 ± 19.7 ^ab^	18.0 ± 0.9 ^a^	12
	13	2.9 ± 0.5 ^abc^	1.6 ± 0.4 ^a^	3.0 ± 0.7 ^ab^	16.3 ± 1.5 ^a^	34.4 ± 5.5 ^a^	17.4 ± 1.7 ^a^	12

^1^ TCC: total chlorophyll content; ^2^ TCarC: total carotenoid content; ^3^ TAC: total anthocyanin content (expressed as CyEs, cyanidin-3,5-O-diglucosid equivalents); ^4^ TPC: total phenolic content (expressed as GAEs, gallic acid equivalents); ^5^ FC: flavonoid content (expressed as CEs, catechin equivalents, or QEs, quercetin equivalents). Notes: treatments: P represents thermopriming, C control conditions (non-primed or non-stressed groups), and S salt stress (e.g., C–C–C: control conditions (non-primed) instead of priming conditions, non-stressed (C) at the time of the first salt stress, and non-stressed (C) at the time of the second salt stress). The different letters indicate significant differences (ANOVA and EMMs post hoc; α = 0.05) between groups in the same week after priming of the corresponding parameter. n corresponds to the sample size of the previous parameters.

**Table A7:** Leaf compounds displayed as mean and standard deviation in mature tomato leaves differentiated for the eight treatments weeks after priming (WAP).

Treatment	WAP	TCC ^1^	TAC ^2^	TPC ^3^	FC_Catechin_ ^4^	FC_Quercetin_ ^4^	n
		[µg mg^−1^ DM^−1^]	[µg CyEs mg^−1^ DM^−1^]	[µg GAEs mg^−1^ DM^−1^]	[µg CEs mg^−1^ DM^−1^]	[µg QEs mg^−1^ DM^−1^]	
C–C–C	3	4.0 ± 0.4 ^a^	2.2 ± 0.4 ^b^	6.2 ± 0.5 ^a^	18.9 ± 1.5 ^b^	12.8 ± 0.6 ^a^	8
	4	4.1 ± 0.2 ^b^	2.3 ± 0.4 ^bc^	7.7 ± 1.9 ^a^	19.0 ± 4.3 ^abc^	12.7 ± 0.3 ^ab^	12
	5	4.0 ± 0.2 ^c^	4.8 ± 0.7 ^c^	6.4 ± 0.5 ^a^	17.6 ± 2.3 ^bcd^	12.9 ± 1.0 ^a^	12
	9	5.3 ± 0.8 ^a^	7.4 ± 1.2 ^a^	8.8 ± 1.2 ^a^	35.5 ± 26.4 ^a^	16.0 ± 1.1 ^ab^	12
	13	5.7 ± 0.7 ^a^	8.1 ± 1.4 ^a^	9.6 ± 1.1 ^a^	26.4 ± 5.8 ^a^	18.1 ± 2.4 ^a^	12
	18	4.0 ± 0.7 ^a^	5.7 ± 0.7 ^a^	9.3 ± 1.1 ^a^	16.0 ± 3.0 ^a^	16.5 ± 1.4 ^a^	12
C–C–S	4	4.1 ± 0.3 ^b^	2.4 ± 0.3 ^bc^	7.8 ± 1.7 ^ab^	20.2 ± 4.2 ^bc^	14.0 ± 2.3 ^bc^	12
	5	3.9 ± 0.2 ^c^	3.2 ± 1.0 ^b^	6.5 ± 0.7 ^a^	14.9 ± 1.6 ^a^	17.3 ± 3.1 ^c^	12
	9	5.0 ± 0.7 ^a^	7.5 ± 1.0 ^a^	8.6 ± 0.6 ^a^	33.1 ± 24.1 ^a^	15.7 ± 1.3 ^ab^	12
	13	5.3 ± 0.8 ^a^	7.9 ± 1.1 ^a^	9.4 ± 1.2 ^a^	23.2 ± 4.9 ^a^	17.5 ± 2.2 ^a^	12
	18	3.8 ± 0.8 ^a^	5.6 ± 1.3 ^a^	9.6 ± 1.3 ^a^	16.1 ± 3.2 ^a^	16.4 ± 2.3 ^a^	12
C–S–C	3	3.9 ± 0.3 ^a^	1.4 ± 0.4 ^a^	7.5 ± 0.9 ^c^	15.6 ± 2.9 ^a^	16.4 ± 1.4 ^b^	8
	4	4.0 ± 0.2 ^ab^	2.1 ± 0.4 ^abc^	10.6 ± 1.7 ^cd^	14.6 ± 1.5 ^a^	15.6 ± 1.8 ^cd^	12
	5	3.9 ± 0.4 ^c^	2.7 ± 0.9 ^ab^	8.1 ± 0.9 ^cd^	16.7 ± 0.9 ^abc^	18.5 ± 1.8 ^c^	12
	9	4.7 ± 1.0 ^a^	6.5 ± 1.3 ^a^	8.7 ± 1.3 ^a^	32.5 ± 20.0 ^a^	15.7 ± 2.4 ^ab^	12
	13	5.3 ± 0.4 ^a^	7.5 ± 0.9 ^a^	8.7 ± 1.1 ^a^	24.3 ± 3.0 ^a^	17.5 ± 2.0 ^a^	12
	18	3.5 ± 0.7 ^a^	5.3 ± 1.2 ^a^	10.6 ± 2.4 ^a^	16.2 ± 2.5 ^a^	17.2 ± 2.5 ^a^	12
C–S–S	4	4.1 ± 0.3 ^b^	2.5 ± 0.4 ^c^	11.2 ± 1.8 ^cd^	17.6 ± 1.3 ^ab^	16.3 ± 1.4 ^d^	12
	5	3.3 ± 0.6 ^ab^	2.9 ± 0.9 ^ab^	9.2 ± 1.1 ^de^	17.2 ± 1.4 ^abcd^	16.2 ± 1.9 ^bc^	12
	9	5.0 ± 0.8 ^a^	7.0 ± 1.4 ^a^	9.0 ± 1.1 ^a^	32.8 ± 20.9 ^a^	16.3 ± 1.7 ^ab^	12
	13	5.3 ± 0.6 ^a^	7.7 ± 0.9 ^a^	10.0 ± 1.8 ^a^	23.5 ± 2.2 ^a^	17.7 ± 1.9 ^a^	12
	18	4.3 ± 0.6 ^a^	6.2 ± 1.0 ^a^	10.4 ± 1.5 ^a^	17.3 ± 2.8 ^a^	17.7 ± 1.8 ^a^	12
P–C–C	3	3.7 ± 0.3 ^a^	1.7 ± 0.3 ^a^	6.4 ± 0.7 ^ab^	17.8 ± 1.6 ^b^	12.4 ± 0.6 ^a^	8
	4	3.6 ± 0.3 ^a^	1.9 ± 0.3 ^ab^	7.2 ± 1.8 ^a^	15.9 ± 3.2 ^ab^	10.8 ± 0.7 ^a^	12
	5	3.6 ± 0.3 ^bc^	3.0 ± 1.1 ^ab^	6.2 ± 0.6 ^a^	16.1 ± 2.5 ^abc^	12.7 ± 0.5 ^a^	12
	9	5.0 ± 0.7 ^a^	7.3 ± 1.2 ^a^	8.0 ± 1.0 ^a^	35.8 ± 23.5 ^a^	14.8 ± 1.6 ^a^	12
	13	5.7 ± 0.4 ^a^	8.1 ± 1.3 ^a^	8.9 ± 0.9 ^a^	27.8 ± 5.0 ^a^	17.8 ± 2.1 ^a^	12
	18	4.1 ± 0.6 ^a^	6.0 ± 0.8 ^a^	9.8 ± 0.9 ^a^	17.4 ± 2.5 ^a^	17.4 ± 1.3 ^a^	12
P–C–S	4	3.7 ± 0.4 ^ab^	2.0 ± 0.3 ^ab^	8.1 ± 2.0 ^ab^	17.1 ± 5.7 ^ab^	11.2 ± 0.8 ^a^	12
	5	3.6 ± 0.4 ^bc^	3.0 ± 0.9 ^ab^	6.8 ± 1.0 ^ab^	15.7 ± 2.6 ^ab^	13.3 ± 2.0 ^a^	12
	9	4.9 ± 1.0 ^a^	6.7 ± 1.4 ^a^	8.7 ± 0.9 ^a^	34.8 ± 18.0 ^a^	15.2 ± 1.2 ^a^	12
	13	5.3 ± 0.8 ^a^	7.3 ± 0.9 ^a^	9.6 ± 1.8 ^a^	27.7 ± 6.7 ^a^	16.9 ± 2.2 ^a^	12
	18	4.0 ± 0.8 ^a^	5.9 ± 0.9 ^a^	9.8 ± 1.0 ^a^	17.6 ± 4.2 ^a^	16.9 ± 1.6 ^a^	12
P–S–C	3	3.8 ± 0.4 ^a^	1.6 ± 0.3 ^a^	7.4 ± 0.9 ^bc^	15.1 ± 3.1 ^a^	15.4 ± 1.9 ^b^	8
	4	3.9 ± 0.3 ^ab^	2.1 ± 0.3 ^abc^	9.9 ± 2.2 ^bc^	15.8 ± 2.7 ^ab^	16.5 ± 1.6 ^d^	12
	5	3.5 ± 0.4 ^bc^	3.2 ± 0.8 ^b^	7.7 ± 0.7 ^bc^	19.9 ± 2.7 ^d^	17.3 ± 2.3 ^c^	12
	9	5.0 ± 0.4 ^a^	7.2 ± 0.9 ^a^	8.9 ± 1.1 ^a^	35.6 ± 22.2 ^a^	15.7 ± 2.3 ^ab^	12
	13	5.5 ± 0.7 ^a^	8.0 ± 1.2 ^a^	10.1 ± 1.2 ^a^	26.7 ± 5.0 ^a^	18.1 ± 2.5 ^a^	12
	18	3.9 ± 0.9 ^a^	5.7 ± 1.4 ^a^	10.2 ± 1.4 ^a^	18.1 ± 5.8 ^a^	16.8 ± 1.8 ^a^	12
P–S–S	4	3.6 ± 0.5 ^a^	1.9 ± 0.5 ^a^	12.3 ± 1.7 ^d^	23.0 ± 5.3 ^c^	15.9 ± 2.1 ^d^	12
	5	2.9 ± 0.7 ^a^	2.1 ± 0.7 ^a^	9.7 ± 1.5 ^e^	18.7 ± 2.1 ^cd^	14.4 ± 2.3 ^ab^	11
	9	4.9 ± 0.8 ^a^	6.5 ± 1.0 ^a^	8.4 ± 0.7 ^a^	37.9 ± 21.4 ^a^	18.1 ± 4.5 ^b^	12
	13	5.4 ± 0.7 ^a^	7.3 ± 0.8 ^a^	9.3 ± 1.5 ^a^	26.6 ± 4.8 ^a^	17.4 ± 1.5 ^a^	12
	18	4.1 ± 0.6 ^a^	5.9 ± 0.7 ^a^	10.5 ± 1.3 ^a^	18.3 ± 3.5 ^a^	18.1 ± 1.7 ^a^	12

^1^ TCC: total chlorophyll content; ^2^ TAC: total anthocyanin content (expressed as CyEs, cyanidin-3,5-O-diglucosid equivalents); ^3^ TPC: total phenolic content (expressed as GAEs, gallic acid equivalents); ^4^ FC: flavonoid content (expressed as CEs, catechin equivalents, or QEs, quercetin equivalents). Notes: treatments: P represents thermopriming, C control conditions (non-primed or non-stressed groups), and S salt stress (e.g., C–C–C: control conditions (non-primed) instead of priming conditions, non-stressed (C) at the time of the first salt stress, and non-stressed (C) at the time of the second salt stress). The different letters indicate significant differences (ANOVA and EMMs post hoc; α = 0.05) between groups in the same week after priming of the corresponding parameter. n corresponds to the sample size of the previous parameters.
